# Endocan as a potential marker in diagnosis and predicting disease severity in COVID-19 patients: a promising biomarker for patients with false-negative RT-PCR

**DOI:** 10.48101/ujms.v127.8211

**Published:** 2022-01-24

**Authors:** Esra Laloglu, Handan Alay

**Affiliations:** a Department of Medical Biochemistry, Faculty of Medicine, Ataturk University, Erzurum, Turkey; b Department of Infectious Diseases and Clinical Microbiology, Faculty of Medicine, Ataturk University, Erzurum, Turkey

**Keywords:** Biomarker, COVID-19, endocan, reverse transcription polymerase change reaction, SARS-CoV-2

## Abstract

**Background:**

Endothelial-specific molecule 1 (endocan) has emerged as an inflammatory biomarker in recent years. The purpose of this study was to investigate the diagnostic value of serum endocan levels in the prediction of COVID-19 disease among patients with a false-negative reverse transcription polymerase change reaction (RT-PCR) test, and also to determine its correlation with the clinical severity of the disease.

**Methods:**

Thirty patients with positive RT-PCR results and 30 with false-negative RT-PCR results, both with suspected COVID-19 in terms of clinical, radiological, and laboratory findings, were included in the study. Thirty healthy controls were also enrolled.

**Results:**

Serum endocan levels were estimated to be 821.8 ± 99.3 pg/mL in COVID-19 RT-PCR (+) patients, 803.9 ± 97.0 pg/mL in RT-PCR false (–) patients with suspected COVID-19, and 382.9 ± 37.5 pg/mL in the control group. No significant difference was observed between RT-PCR (+) and RT-PCR false (–) patients (*P* = 0.68). However, serum endocan levels differed significantly between patient groups and control group (*P* < 0.05). With a cut-off value of 444.2 pg/mL serum endocan levels differentiated COVID-19 cases from healthy individuals with 92% sensitivity and 80% specificity. Moreover, a significant positive correlation was observed between serum endocan levels and clinical severity (*P* < 0.01, *r* = 0.94).

**Conclusions:**

There is a need for different laboratory markers capable of assisting diagnosis and showing COVID-19 infection in suspected COVID-19 RT-PCR false-negative patients. Endocan levels can be used as an assistant blood test for identifying COVID-19 patients with false-negative RT-PCR tests and in determining the clinical severity of the disease.

## Introduction

The respiratory tract infection caused by severe acute respiratory syndrome coronavirus 2 (SARS-CoV-2) was first detected in the city of Wuhan, China, at the end of 2019. The virus subsequently spread rapidly, affecting the entire world, leading to the deaths of thousands. The World Health Organization declared a pandemic in March 2020, and named the SARS-CoV-2 infection coronavirus disease 2019 (COVID-19) (1, 2).

The virus attaches to the angiotensin converting enzyme-2 (ACE-2) receptor through the receptor-binding region of the spike proteins on the membrane and enters the mammalian cell by initiating the membrane fusion. The virus frequently settles in the pulmonary parenchyma. Severe pneumonia and/or acute respiratory distress syndrome (ARDS) will cause vasoconstriction, bronchoconstriction, increased vascular permeability, inflammation, and fibrosis. Even subsequent pulmonary insufficiency occurs in many patients infected with the activation of the ACE-2 enzyme (3, 4). In some patients, the severity of the inflammatory response to the virus continues to increase, resulting in systemic inflammation. This situation, generally known as the cytokine storm, can lead to damage in distant organs (3).

The real-time reverse transcription polymerase change reaction (RT-PCR) test is recognized as the gold standard in the diagnosis of the disease. The early diagnosis and treatment of COVID-19, with its high risk of mortality and morbidity, especially in the at-risk group, are important in preventing complications and of placing such individuals to prevent transmission. Simple, low-cost tests, with high sensitivity and specificity, capable of being studied in serum or plasma, therefore, need to be established for the diagnosis and follow-up of the disease. One of the molecules investigated for that purpose is endothelial cell-specific molecule-1 (ESM-1 or endocan).

Endocan, a member of the proteoglycan family, is primarily produced in kidney, lung, and gastrointestinal tract endothelial cells as a response to proangiogenic growth factors and proinflammatory cytokines (5). It has been shown that endocan may play a potential role in the regulation of cell adhesion, tumor spread, and inflammatory events (6).

The purpose of this study was to determine the diagnostic value of serum endocan levels in patients infected with the SARS-COV-2 and their association with the clinical severity of the disease.

## Materials and methods

### Study population

The study commenced following receipt of approval from the Ataturk University Medical Faculty Ethical Committee (No.B.30.2.ATA.0.01.00/211). Sixty patients presenting to the Infectious Diseases and Clinical Microbiology Clinic between May and October 2020 with symptoms such as cough, fever, muscle pain, sore throat, and nasal discharge and diagnosed with COVID-19 through radiological and laboratory tests in addition to physical examination were included. A SARS-CoV-2 (2019-nCoV) qPCR detection kit (Bio-Speedy Bioeksen) was used to detect the epidemic virus ‘SARS-CoV-2 (2019-nCoV)’ responsible for COVID-19. The kit was applied to nucleic acid isolates from nasopharyngeal and oropharyngeal swab specimens. Radiological evaluations were performed by thoracic computed tomography. Complete blood count, alanine aminotransferase (ALT), aspartate aminotransferase (AST), gamma-glutamyl transferase (GGT), lactate dehydrogenase (LDH), glucose, erythrocyte sedimentation rate (ESR), C-reactive protein (CRP), troponin-I, procalcitonin, D-dimer, and ferritin levels were measured as laboratory diagnostic tests.

The patient group was divided into two subgroups. Group 1 consisted of 30 patients with positive RT-PCR test results and with physical examination, radiological, and other laboratory findings compatible with COVID-19 on admission. Thirty patients with negative RT-PCR test results and with physical examination, radiological, and other laboratory findings compatible with COVID-19 on admission constituted Group 2. The RT-PCR test results of the patients in this group were evaluated as false negative. The second RT-PCR test results obtained within 24–48 h from Group 2 were all positive. As the RT-PCR test-positive COVID-19 patients (Group 1), Group 2 patients were also placed under quarantine and started on the requisite medical treatment. Thirty healthy individuals with normal physical examination findings and routine laboratory tests constituted the control group. The control group had come for health check-up, a comprehensive health examination in the outpatient department. These were SARS‐CoV‐2 RT‐PCR‐negative at the time of inclusion in the study. All three groups were informed about the study and were enrolled after providing informed consent. The severity of the disease was classified as uncomplicated, mild to moderate pneumonia, and severe pneumonia. Critical cases in need of intensive care were treated according to the adult COVID-19 patient diagnosis and treatment guidelines published by the Turkish Ministry of Health and literature (7, 8). Exclusion criteria included the presence of any malignancy, hypertension, cardiovascular disease, diabetes mellitus, chronic kidney disease, or acute or chronic inflammatory disease.

### Blood specimens

Blood specimens collected for routine tests from the patient and control groups before the start of any medical treatment were left for 10–20 min in tubes in a vertical position for coagulation. They were then centrifuged at +4°C for 15 min at 4,000 rpm. The sera specimens obtained were aliquoted and placed into a deep freeze at –80˚C until analysis.

### Analyte assay techniques

Serum endocan levels were measured by an enzyme-linked immunosorbent assay (ELISA) method with a human endocan ELISA kit as recommended by the manufacturer. The kit measurement range was 15–1,000 pg/mL. The intra-assay coefficient of variation (CV) of 4.8% and an inter-assay CV of 6.2%. Serum glucose (mg/dL), CRP (mg/L), AST (U/L), ALT (U/L), GGT (U/L), and LDH (U/L) levels were measured on a Beckman Coulter AU5821 device. Ferritin (ng/ml) and troponin-I (ng/L) levels were measured on a Beckman Coulter DxI800 device, and procalcitonin (ng/ml) levels on a Roche Cobas 6000 device with commercial kits. ESR values were investigated using the Westergren method on a StaRRsed device, results being expressed as mm/h. White blood cell (WBC), lymphocyte, and neutrophil counts were estimated on a Sysmex XN-9000 (Japan) device, and the results were expressed as cells/μL. D-dimer levels (ng/ml) were measured on a Radiometer AQT90 FLEX device.

### Statistical analysis

Data were analyzed using SPSS 20.0 for Windows software (SPSS Inc., IL, USA). Descriptive statistics were shown as number and % for categorical variables and as mean ± standard deviation for numerical variables. The normality of distribution was evaluated using visual (histograms, probability plots) and analytical methods (Kolmogorov-Smirnov/Shapiro–Wilk test). The study groups were compared using one-way analysis of variance (ANOVA), and the significance of differences between groups was evaluated using the post-hoc Tukey test. The chi-squared (χ^2^) was applied for the statistical analysis of differences in gender distributions between the groups. The relationships between endocan levels and laboratory tests were evaluated using Pearson’s correlation. The relationships between disease severity and serum levels of endocan, ESR, CRP, ferritin, and D-dimer parameters were evaluated using Spearman’s correlation. The ROC curve, an expression of the predictive power of a specific method, was used to calculate the sensitivity, specificity, area under the curve (AUC), and cut-off value of serum endocan. *P* values <0.05 were considered to be statistically significant.

## Results

The mean age of the 30 cases included in Group 1 was 53.9 ± 16.4 years compared with 59.9 ± 17.1 years in the 30 initially PCR false (-) cases and 54.5 ± 14.4 years in the healthy individuals. Statistical analysis (ANOVA) revealed no significant difference between the groups in terms of age (*P* = 0.29).

Twelve (40%) of the patients in Group 1 were women and 18 (60%) were men. Women constituted 14 (46.7%) of the cases in Group 2, and men 16 (53.3%), while in the control group 13 (43.3%) were women and 17 (56.7%) were men. Analysis revealed no significant difference between the groups in terms of gender distribution (X^2^ test, *P* = 0.83).

Laboratory test results in the diagnosis of COVID-19 are presented in Table 1. Radiological examinations of the PCR (+) and PCR false (–) Covid-19 patients, excluding the uncomplicated patient group, revealed different levels of involvement in the lungs consistent with the disease (Table 2). When the cases in Groups 1 and 2 were classified based on the clinical severity of disease, 13 (21.7%) were uncomplicated, 24 (40%) were classified as mild to moderate pneumonia, 11 (18.3%) as severe pneumonia, and 12 (20%) as critical. When the patient groups were evaluated together, there was a positive correlation between the clinical severity of the disease and serum endocan, D-dimer, CRP, ferritin, and ESR values (*r* = 0.94, *r* = 0.85, *r* = 087, *r* = 0.67, and *r* = 0.77, respectively, *P* < 0.01 for all).

**Table 1:** Laboratory tests.

Parameter	Group 1 (*n* = 30)	Group 2 (*n* = 30)	Control (*n* = 30)	*P*
Aspartate aminotransferase (U/L)	44.6±28.7	39.21±27.2	26.8±8.6	0.801^a^ 0.091^b^ 0.367^c^
Alanine aminotransferase (U/L)	36.9±25.1	28.86±23.4	27.5±10.9	0.492^a^ 0.370^b^ 0.981^c^
Lactate dehydrogenase (U/L)	426.62±195.7	420.21±188.1	192.2±42.5	0.108^a^ **<0.001^b,c^**
Gamma-glutamyl transferase (U/L)	45.8±40.2	54.5±41.75	27.6±10.9	0.620^a^ 0.121^b^ **0.023^c^**
Glucose (mg/dL)	122.6±55.8	127.0±51.0	82.9±8.3	0.998^a^ **<0.05^b,c^**
Erythrocyte sedimentation rate (mm/h)	33.19±25.1	39.9±24.5	8.6±3.5	0.389^a^ **<0.001^b,c^**
C-reactive protein (mg/L)	103.14±70.2	99.2±61.4	1.9±1.2	0.255^a^ **<0.001^b,c^**
Procalcitonin (ng/mL)	0.09±0.1	0.06±0.05	0.06±0.07	0.624^a^ 0.651^b^ 0.999^c^
Ferritin (ng/mL)	704.9±687.7	733.2±660.5	211.6±60.7	0.981^a^ **<0.05^b, c^**
Troponin I (ng/L)	4.3±3.8	4.9±4.2	2.9±1.7	0.864^a^ 0.4^b^ 0.222^c^
White blood cell (cells/ μL)	5,831.2±1,876.5	5,357.2±1,469.3	7,529.4±1,563.4	0.479a **<0.05^b, c^**
Neutrophil (cells/ μL)	3,362.6±1,781.9	3,106.9±1,467.3	4,098.7±1,039.2	0.725^a^ **<0.05^b, c^**
Lymphocyte (cells/ μL)	1,301.5±522.1	1,252.7±519.8	1,963.7±671.2	0.528^a^ **<0.001^b, c^**
D-dimer (ng/mL)	1,515.2±977.5	1,541.5±1,068.8	208.9±89.5	0.93^a^ **0.002^b, c^**
Endocan (pg/mL)	821.9± 99.4	803.9 ± 97.0	382.9 ± 37.6	0.68^a^ **<0.05^b, c^**

Values are presented as mean ± standard deviation.

a , between Groups 1 and 2.

b , between Group 1 and the control group.

C , between Group 2 and the control group.

**Table 2:** Radiological evaluation of pulmonary involvements.

Severity (*n*)	Normal	Ground glass opacity	Consolidation	Both of ground glass and consolidation	Unilateral/bilateral pulmonary lesions	Paving stone sign
Uncomplicated (*n* = 13)	13	-	-	-	**-**	**-**
Mild to moderate (*n* = 24)	-	15	10	8	**14 / 10**	**6**
Severe pneumonia (*n* = 11)	-	5	6	4	**- / 11**	**7**
Critical level (*n* = 12)	-	6	8	4	**- / 12**	**6**

**Figure 1:**
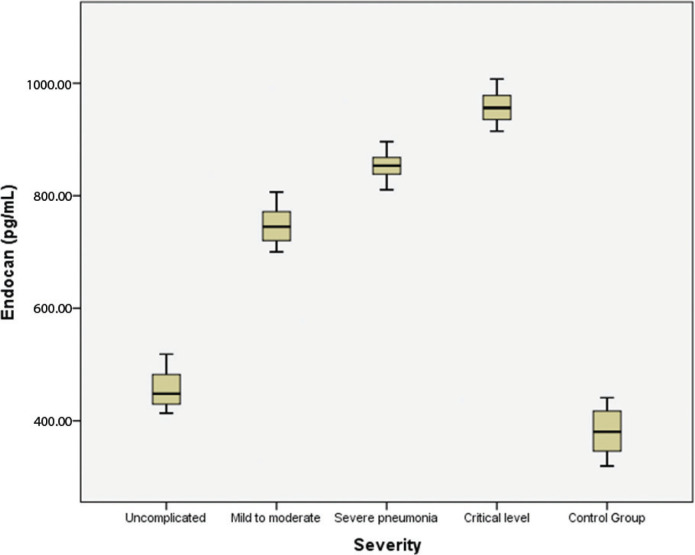
A box-plot chart showing serum endocan levels in different COVID-19 disease severity groups.

**Figure 2:**
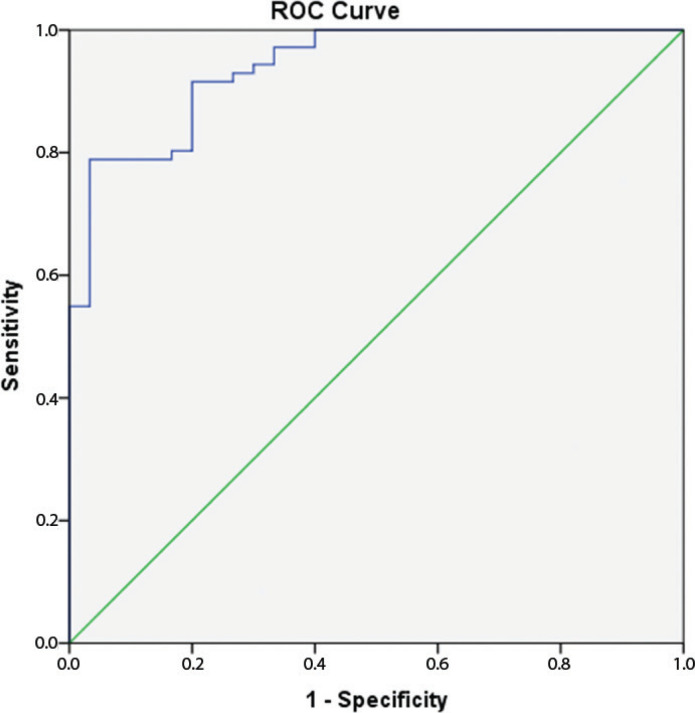
Determination of the diagnostic sensitivity and specificity of serum endocan levels in patients with COVID-19 by ROC curve analysis. ROC: receiver-operating characteristic curve.

Comparisons revealed no significant difference between the serum endocan levels of the PCR (+) and PCR false (–) COVID-19 patients (*P* = 0.68). However, there were significant differences in serum endocan levels between the patient groups and the control group (*P* < 0.05 for both; Table 1). Serum endocan levels in the groups established on the basis of disease severity are shown in Table 3 and Figure 1. With a cut-off value of 444.2 pg/mL, serum endocan levels exhibited 92% sensitivity and 80% specificity in differentiating COVID-19 cases from healthy individuals, with a positive predictive value of 82% and a negative predictive value of 91% (AUC = 0.94, *P* < 0.001, 95% confidence interval 0.89–0.98, likelihood ratio for negative result = 10, likelihood ratio for positive result = 4.6) (Figure 2).

**Table 3:** Disease severity and serum endocan levels.

	Uncomplicated (n = 13)	Mild to moderate(*n* = 24)	Severe pneumonia (*n* = 11)	Critical level (*n* = 12)	*P*
**Endocan (pg/mL)**	556.5± 32.9	717.2 ± 36.2	865.4± 27.6	925.4±39.1	<0.001*

Values expressed as mean ± standard deviation.

* , comparison of all groups

The correlation coefficient between serum endocan levels and laboratory tests frequently employed in the diagnosis of COVID-19 when the patient and control groups were evaluated together is presented in Table 4. LDH, ferritin, CRP ESR, and D-dimer values, which occupy particularly important places in diagnosis, treatment, and prognosis, were significantly and positively correlated with serum endocan levels (*r* = 0.71, *r* = 0.67, *r* = 0.87, *r* = 0.77, and *r* = 085 respectively. *P* < 0.001 for all).

**Table 4:** Correlation between serum endocan levels and laboratory tests.

		Aspartate aminotransferase (U/L)	Alanine aminotransferase (U/L)	Gamma-glutamyl transferase (U/L)	Troponin-I (ng/L)	Glucose (mg/dL)	Procalcitonin (ng/mL)
**Endocan (pg/mL)**	Correlation coefficient* **(*r*)**	0.486	0.183	0.464	0.483	0.399	0.313
	***P***	<0.001	0.067	<0.001	<0.001	<0.001	0.001

* , Pearson’s correlation.

## Discussion

Laboratory tests currently constitute a mean 70% of the process of diagnosis, treatment, and follow-up of diseases. During diagnosis, clinicians consider the patient’s family history, symptoms, examination findings, and laboratory tests. As laboratory tests generally yield numerical data, they are of particular assistance to the clinician in diagnosis, compared with subjective data, such as history and physical examination findings. Such tests occupy an indisputable place in the diagnosis, treatment, and follow-up of COVID-19 disease. Since the disease has become a global pandemic, its adverse impacts have been felt in many spheres, such as the economy, health, and education. Prompt diagnosis and quarantining of patients are, therefore, important in reducing its transmission. Laboratory tests employed during diagnosis, treatment, and follow-up are also of use to the clinician in disease classification, predicting patients with low or high mortality risks, and in monitoring the therapeutic process. Severe complications, such as ARDS, disseminated intravascular coagulation, and multi-organ failure, can be seen in patients with cytokine storm (3). Several laboratory tests are used to in order to identify these patients beforehand and to provide the appropriate treatment in a timely manner.

The RT-PCR test, the gold standard in detecting diseases of viral origin for the last 20 years, is also employed in the diagnosis of COVID-19 disease. Although the test has high sensitivity and specificity in the laboratory setting, according to data from the Wuhan outbreak, its sensitivity in clinical practice is between 31 and 71% (9).

Several variables, including the amount and type of specimen, its transportation, and the stage of disease during which it was collected, affect the sensitivity of the test. Interpretation of the RT-PCR test is complicated, and the method is costly, and it is therefore best suited to central reference laboratories. Completion of the test takes 4–6 h; however, due to logistical obstacles such as specimen collection, transportation, and queuing, the results typically are revealed after 12–24 h (10, 11). Low sensitivity due to pre-analytical factors and difficulties in accessing the test mean that greater use is made of X-rays and computed tomography in diagnosis. Despite their great contribution to diagnosis and disease management, the principal obstacles to the frequent and widespread use of imaging methods are exposure to radiation and the possibility of contributing to the spread of the disease. The main transmission route in radiology units is surface contamination following droplet spread (9, 12).

The RT-PCR test has been reported to be capable of resulting positive in only 30–60% of cases of suspected COVID-19 based on clinical and other laboratory or radiological findings. In addition to delaying the initiation of treatment due to late diagnosis, false negative results can contribute to the spread of the disease as these patients cannot be isolated. Diagnosed patients being erroneously reported as ‘negative’ during the convalescence period can lead to the spread of infection in the community as quarantine and isolation conditions are cut short while these patients are still infectious (9, 12).

Because of these reasons, there is a need for capable, alternative tests to the RT-PCR test in the diagnosis of COVID-19 disease. Such tests have to produce rapid results in emergency conditions, and should be of low cost, simple to analyze and interpret, with high sensitivity and specificity, and capable of being studied in blood samples. It is now a matter of urgency to find different laboratory markers capable of showing COVID-19 infection, particularly in suspected, RT-PCR-false negative patients, and for assisting diagnosis to halt the pandemic. In present study, no statistically significant difference in serum endocan levels was observed among RT-PCR-positive and RT-PCR-false-negative COVID-19 patients. Therefore, endocan seems as a potential supplementary test for use in the diagnosis of COVID-19.

Numerous publications have shown an association between cytokines and endocan, with its potential role in inflammation. Lee et al. (13) reported high blood and urine endocan levels in patients with renal transplantation and developing microvascular inflammation. These authors suggested that endocan may be capable of use as a potential marker in the development of microvascular inflammation in renal transplant patients. Mertoglu et al. (14) reported that increased endocan levels in fibromyalgia cases support the mechanisms of inflammation and endothelial dysfunction in the pathophysiology of fibromyalgia, and that endocan may even be an important marker in the diagnosis of fibromyalgia. Ozdemir et al. (15) suggested that epileptogenic zone endocan levels increased in patients with epilepsy, and that endocan can, therefore, be employed as an inflammation index in these patients.

Procalcitonin levels are used as a routine laboratory test in the diagnosis and follow-up of patients with sepsis. One of the studies of patients with systemic inflammatory response syndrome described increased endocan and procalcitonin levels. The sensitivity and specificity of endocan and procalcitonin levels for patients diagnosed with sepsis were 87.2 and 81.8, and 85.9 and 81.8%, respectively. The sensitivity and specificity of endocan and procalcitonin levels for ‘septic death prediction’ were 95.7 and 70.9, and 65.2 and 78.2%, respectively. The authors, therefore, suggested that endocan was a more useful clinical marker than procalcitonin for diagnosis and predicting prognosis (16). In this study, serum endocan levels were higher in the COVID-19 patients compared with the control group. Moreover, there was a significant positive correlation between endocan levels and the inflammatory markers CRP, sedimentation and ferritin, useful guides in the diagnosis, treatment, and follow-up of COVID-19. This finding supports the idea that endocan is a potential inflammatory marker in the diagnosis of COVID-19.

The ACE-2 receptor used by the SARS-COV-2 when infecting cells is expressed at high levels in the lung (17). Severe pneumonia and/or ARDS are therefore found in 20–30% of patients infected with SARS-COV-2 (4). The morbidity and mortality of COVID-19 most frequently emerge in association with acute viral pneumonia and ARDS (18). High endocan levels have been reported in patients with ventilator-associated pneumonia (VAP). Elevated endocan levels have been linked to the development of VAP, and that this can be used as a screening test (19). Another study reported increased serum endocan levels in severe sepsis patients requiring mechanical ventilation in the intensive care unit. The authors proposed that endocan can be employed as a guide in the early period for identifying the mechanical ventilation requirements of patients with severe sepsis (20). High endocan levels have also been detected in patients followed up in the intensive care unit due to ARDS. Therefore, it has been suggested that endocan can be used in the evaluation of disease severity in patients with ARDS (21). One of the studies of COVID-19 patients in intensive care requirements reported high endocan levels and concluded that these might be useful in evaluating the prognosis in hospitalized patients (22).

Radiological examination of the patients included in this study (excluding the uncomplicated cases) revealed varying degrees of pulmonary involvement, bilateral or unilateral involvement, ground-glass opacity, consolidation, and a crazy-paving pattern. The parameter most strongly correlated with clinical severity in this study was endocan. Serum endocan levels increased significantly in line with disease severity, being highest in the critical patients.

One of the important complications developing in patients with COVID-19 is thrombosis. Increased inflammation, hypoxemia, immobilization, and increased intravascular inflammation in patients diagnosed with COVID-19 can lead to thrombosis in both the venous and arterial systems (23, 24). One of the study of patients with deep vein thrombosis (DVT) concluded that markers synthesized from the endothelium, such as endocan, may be useful in the evaluation of DVT (25). A significant positive correlation was also observed between endocan and D-dimer levels in this study. This also suggests that endocan may be a useful marker in the evaluation of thrombosis development in patients with COVID-19.

The principal limitation of this study was that no false-positive population has been encountered. The specificity (>95%) of the RT-PCR test is higher than its sensitivity (70%). Therefore, a positive RT-PCR test is more significant than a negative (26, 27). Also, another control group with similar symptoms and signs related to non-COVID-19 diseases will clarify whether high endocan levels are specific to COVID-19 or inflammation due to any other reason. Further studies involving larger patient numbers are now needed to confirm the role of endocan in identifying the COVID-19 patients with false-negative RT-PCR tests.

In conclusion, our results (higher endocan levels in RT-PCR false (–) patients than those in the control group but similar endocan levels to those in the RT-PCR (+) patients, presence of a significant association between clinical severity and endocan levels, the high diagnostic and predictive values, easy to analysis, the fact that it can be studied in routine laboratories, and that it yields rapid results) suggest that endocan may be a useful marker in the diagnosis, and follow-up of treatment in patients with COVID-19.

## Data Availability

The data that support the findings of this study are available from the corresponding author upon reasonable request.
